# Sustainable adoption of artificial intelligence and the Metaverse in higher education: an environmental, social, and governance–based analysis of pedagogical innovation and perceived student learning outcomes

**DOI:** 10.3389/frai.2026.1738730

**Published:** 2026-03-04

**Authors:** Jehad Alqurni

**Affiliations:** Department of Educational Technologies, College of Education, Imam Abdulrahman Bin Faisal University, Dammam, Saudi Arabia

**Keywords:** artificial intelligence, digital pedagogical innovation, ESG framework, higher education, Metaverse, student learning outcomes, sustainable adoption

## Abstract

The rapid convergence of Artificial Intelligence (AI) and Metaverse technologies is reshaping the higher education landscape by enabling immersive, personalized, and adaptive learning experiences. However, the long-term sustainability of such innovations remains uncertain without addressing environmental, social, and governance (ESG) considerations. This study develops and empirically validates an ESG-informed framework for Sustainable AI–Metaverse Adoption (SAAM) in higher education. A quantitative research design was employed, collecting data from 280 university students across diverse disciplines through a structured survey. Structural Equation Modeling (SEM-PLS) was applied to assess measurement reliability, convergent and discriminant validity, and to test the proposed hypotheses. The empirical results demonstrate that ESG dimensions exert differential effects on sustainable adoption, with environmental and social factors showing stronger direct associations than governance-related variables. Environmental sustainability, through energy-efficient AI systems, significantly enhances SAAM. Similarly, social dimensions, particularly inclusive AI access and student acceptance, exert robust positive effects on sustainable adoption, whereas faculty readiness influences adoption indirectly. Conversely, governance-related factors exhibit comparatively weaker direct effects: institutional policy support enhances digital infrastructure but does not directly influence SAAM, whereas ethical AI use has a limited impact, reflecting student prioritization of usability over ethics in early stages of adoption. Importantly, the outcomes highlight that SAAM substantially fosters digital pedagogical innovation (DPI) and enhanced student learning outcomes (ESLO), confirming its transformative potential. The study contributes theoretically by integrating ESG principles into technology adoption research, offering a multidimensional lens that enriches the understanding of sustainable digital transformation in higher education. Practically, it provides institutions and policymakers with evidence-based insights to design environmentally conscious, socially inclusive, and governance-supported strategies for AI–Metaverse integration. Future research should expand to cross-cultural contexts, larger samples, and longitudinal designs to validate and generalize these findings.

## 1 Introduction

Higher education has experienced sustained digital transformation over the past two decades; however, the Coronavirus disease 2019 (COVID-19) pandemic marked a decisive acceleration in this process (Zawacki-Richter et al., 2020; Bond et al., 2020). The abrupt transition to remote and hybrid learning environments exposed both the potential and limitations of existing digital education tools, including learning management systems, massive open online courses (MOOCs), and video-conferencing platforms. Prior studies have observed that while these technologies expanded access and flexibility, they also amplified longstanding challenges related to student motivation, digital inequality, and institutional readiness for large-scale digital delivery (Dahri et al., 2025b). These challenges have intensified calls for more advanced, adaptive, and resilient educational technologies that support meaningful learning experiences beyond emergency remote instruction.

Artificial Intelligence (AI) and Metaverse technologies are seen as the future of higher education, as they transform higher education (Rana et al., 2022; Dahri et al., 2024e). The earlier phase of digitization merely transferred offline teaching and learning to an online format. However, with AI and the Metaverse, we are witnessing a structural shift in teaching and learning processes toward immersion and personalization. This convergence creates adaptive learning environments where teaching, learning, and assessment can be adapted to individual learners in real time, which can alter the design and experience of teaching and learning (Joshi and Pramod, 2023). The advancement of education technology offers great potential for enhancing engagement and pedagogy. However, they simultaneously raise pertinent questions related to sustainability, equity, ethics, and governance.

The Metaverse is usually thought of as a continuous and interconnected virtual environment combining VR, XR, virtual worlds, blockchains, and other such infrastructures (Lawson McLean and Lawson McLean, 2024; Yu et al., 2025). Metaverse-based application development is underway in the higher education sector. The medical course can possess immersive simulations to allow students to rehearse clinical and surgical procedures in a controlled environment without risk (Lawson McLean and Lawson McLean, 2024; Yu et al., 2025). Engineering programs incorporate virtual labs and collaborative simulations to offer hands-on experiences regardless of physical infrastructure limitations (Zontou et al., 2024; Pedram et al., 2023). In the same manner, teacher preparation programs are employing classroom simulations with increasing frequency to help pre-service teachers develop teaching strategies and classroom management (Mystakidis, 2022). The diverse educational uses of Metaverse technologies will soon be illustrated in these apps.

AI enables immersive learning and teaching environments by supporting intelligent tutoring systems, real-time learning analytics, adaptive feedback systems, and conversational agents (Almogren et al., 2024; Dahri et al., 2024b). AI-enabled avatars and natural language processing (NLP) systems enrich interaction and communication, enabling learners to receive continuous scaffolding and personalized support throughout their learning experience (Alwakid and Dahri, 2025; Dahri et al., 2025c). According to various studies, students are more engaged and motivated, and less likely to drop out, when enrolled in fully immersive courses than in blended or traditional learning. The adoption of AI–Metaverse in higher education is still at an early stage, with most initiatives being pilot projects. Development of scalable infrastructure, long-term maintenance, and evaluation frameworks that are underdeveloped. This hampers the technologies’ steps toward broader institutionalization contexts (Mystakidis, 2022; Fitrianto and Saif, 2024; Mystakidis et al., 2022).

The use of AI and the Metaverse technologies is not just a technical possibility but also an ethical and sustainable governance issue. Substantial computational resources and large-scale AI systems demand energy and cause significant carbon emissions (Strubell et al., 2020). Consequently, the sustainability of AI-enabled learning environments is being compared to international sustainability frameworks such as the United Nations Sustainable Development Goals (SDGs) (Al-Raeei, 2024). There is an inconsistency in accessing immersive technology and advanced digital infrastructure from a social point of view, which reiterates the socio-economic gap between institutions and regions (Kourtesis, 2024). Using them in assessment and decision-making contexts is fraught with further ethical concerns around data transparency, algorithmic bias, and accountability (Whittlestone et al., 2019). An AI-powered evaluation that is biased can simply game the whole process. Similarly, data collection in immersive environments involves serious and complex issues of privacy and surveillance that require law and governance to regulate (Tang, 2025). Without coherent institutional policies and regulatory frameworks, these challenges will threaten the credibility of educational technologies and worsen discrimination.

A 2025 systematic review shows an increase in the use of generative AI in teaching. Analyzing the use of generative tasks shows that students can develop their creativity, critical thinking, learning autonomy, and prompt literacy. This will occur as teachers use the latest technologies in their teaching and learning process (Qian, 2025). Research on the combination of AI and Metaverse environments indicates that students become more engaged and motivated through interactive learning. However, sustainable adoption may be hindered by infrastructure and algorithmic biases, as well as privacy concerns (Almeman et al., 2025). By 2025, experiential evidence indicates that a generative AI tool is associated with better academic achievement in contexts aligned with the Sustainable Development Goals, primarily through shared metacognition and cognitive offloading. According to various studies employing extended adoption models (e.g., UTAUT-2), Metaverse technology adoption intentions in higher education environments were significantly influenced by hedonic and performance expectancies, thereby further supporting integrative ESG-sensitive frameworks (Iqbal et al., 2025). This recent study underscores the pressing need to consider sustainability, equity, and governance when examining AI–Metaverse adoption in universities.

Environmental, social, and governance (ESG) frameworks provide a more complete approach to these issues. The development of ESG principles in corporate finance to measure sustainability performance is increasingly being utilized in areas such as healthcare, smart cities, and digitalization. In higher education, ESG frameworks assess technological innovations, the authority’s performance and efficiency, as well as environmental sustainability, social inclusion, and institutional accountability. The green criteria stress low-carbon and energy-efficient AI and hardware (Alamandi, 2025; Olanrewaju et al., 2024). Prioritizing social criteria ensures equitable access, inclusivity, ethical engagement, and cultural responsiveness for all in digital learning environments. Acceptable governance is defined as institutional leadership, transparency, accountability, and policy structures that ensure the responsible use of technology. In the adoption of AI–Metaverse in higher education, ESG frameworks remain underexplored.

The extant technology adoption models, which include the Technology Acceptance Model (TAM) and the Unified Theory of Acceptance and Use of Technology (UTAUT), have focused on user perceptions of usefulness and ease of use (Viswanath, 2003). These models advance understanding regarding individual adoption behavior. But, they misrepresent sustainability-oriented considerations that are emerging as key in digital education ecosystems (Siddiqi, 2024; Aslam, 2024). Recent evaluations of Metaverse applications in education indicate that research is fragmented and experimental. They call for integrative frameworks that incorporate dimensions of sustainability and governance (). Furthermore, institutions of higher education are facing increased pressure to pursue technological innovation aligned with ESG principles to achieve SDG-related objectives, especially SDG 4 (Quality Education), SDG 9 (Industry, Innovation, and Infrastructure), and SDG 12 (Responsible Consumption and Production).

This research addresses these gaps by introducing and empirically validating an ESG-guided conceptual model for sustainable adoption of AI–Metaverse in higher education. The framework comprises 10 constructs: energy-efficient AI infrastructure, inclusive access, ethical AI utilization, digital infrastructure, policy support for institutions, faculty preparedness, student acceptance, digital pedagogic innovation, sustainable AI–Metaverse adoption, and improved learning outcomes for students. Employing Structural Equation Modeling (SEM) with survey data from higher education stakeholders, this research explores the causal relationships among these constructs and provides empirical evidence for the framework. This approach makes the following key contributions: it expands ESG frameworks in the context of higher education digital transformation by bringing ESG concerns together with adoption processes. Offers actionable recommendations for HEIs, policymakers, and education technology developers for responsibly and sustainably integrating AI–Metaverse technologies. Provides evidence-based recommendations for matching AI–Metaverse adoption to global sustainability objectives and institutional governance structures. In light of the above gaps, this study pursues the following objectives: a literature review and the testing of a model for responsible AI–Metaverse integration in higher education guided by ESG.

Secondary objectives:

- Analyze empirically ESG constructs that affect adoption using SEM.
- To formulate the policy and operational effects of AI–Metaverse implementation for responsible higher education aligned to the SDGs.

Thus, the research is informed by the following research questions:

1. What ESG factors impact the sustainable deployment of AI–Metaverse technologies in higher education institutions?
2. How do these factors mutually interact to generate adoption dynamics and learning results?
3. How may ESG principles be introduced in higher education institutions such that the integration of AI–Metaverse aligns with global sustainability objectives?

The incorporation of the Metaverse and AI in university education has transformative potential but also poses challenges. Metaverse increases student interaction and performance, while issues of sustainability, equity, and governance emerge. This research examines the catalysts of an ESG-based framework and tests against a theory and actionable knowledge on institutions and policymakers. This ensures that the AI–Metaverse bond benefits university education and enables a sustainable and equitable digital future.

## 2 Literature review

Understanding the adoption of innovative educational technologies such as AI–Metaverse environments requires engagement with established technology acceptance theories while also recognizing their conceptual limitations in sustainability-oriented and immersive learning contexts. Early behavioral models, including the Theory of Reasoned Action (TRA) (Fishbein and Ajzen, 1977) and the Theory of Planned Behavior (TPB) (Ajzen, 1991; Dahri et al., 2024d), emphasized the role of individual attitudes, subjective norms, and perceived behavioral control in shaping behavioral intentions. Building on these foundations, the Technology Acceptance Model (TAM) (Davis, 1989) emerged as a dominant framework in information systems research, highlighting perceived usefulness and perceived ease of use as primary predictors of technology adoption.

Subsequent extensions, such as TAM2 and the Unified Theory of Acceptance and Use of Technology (UTAUT), were developed that add social influence, facilitating conditions, and hedonic motivation, thereby increasing explanatory power. Although widely used, these models were developed in organizational and workplace contexts and are therefore likely to exhibit limited sensitivity to the experiential, sustainability, and social dimensions of AI-to-Metaverse education (Almeman et al., 2025). TAM does not consider environmental costs, ethics, and governance, which play an increasingly important role in digital transformation in higher education (Chatterjee et al., 2023). Along similar lines, UTAUT improves understanding of organizational adoption; however, it takes a limited approach toward other institutional responsibilities related to sustainability and public accountability. Recent extensions, such as UTAUT2 (Venkatesh et al., 2012), introduced consumer orientation through voluntariness and experiential usage. However, these models still do not account for environmental sustainability or ESG. Evidence from higher education contexts suggests that attitudes toward advanced AI technologies are not just dependent on perceived usefulness and ease of use but also on institutional technology readiness and social influence. This indicates the limits of usability-centric adoption models (Sallam, 2025). To address the issue, frameworks such as the unified theory of acceptance and use of Metaverse technology (UTAUMT) attempt to capture those immersive and experiential characteristics of Metaverse platforms. In addition to these developments, social and governance theories, notably the ESG framework, provide a multidimensional focus. This includes the balance among environmental, social inclusion, and governance accountability. Most importantly, these values align precisely with the mission of higher education institutions and global events. For example, Sustainable Development Goal 4: Quality Education. From an application perspective, Metaverse technologies offer immersive simulations, collaborative learning, and role-based interactions that distinguish them from e-learning systems of earlier times. Evidence from empirical studies shows their use across sectors: nursing and medical education employing VR-based simulations in clinical training, which is risk-free (Sanfilippo et al., 2025; Yu et al., 2024); engineering programs using virtual labs to recreate real experimentation; and language learning contexts using immersive environments for contextualized communication practice (Elhambakhsh et al., 2024; Zhou and Divekar, 2025). Giving a sense of immersion and receiving instant feedback in VR makes the environment engaging for students. Artificial Intelligence further enhances the aforementioned affordances through intelligent tutoring systems, adaptive analytics, and natural language processing, enabling personalized learning pathways and predictive support (Alwakid et al., 2025).

Nonetheless, studies show that continued adoption does not rely only on technological affordances. Trust, perceived social value, inclusivity, and institutional support are critical factors enhancing the relevant adoption intentions from a particular perspective. Governance-related constructs perform as organizational-level conditions. For example, institutional policy support, ethical use of AI, and digital infrastructure are conditions for adoption. They do not directly create student acceptance or perceived benefits. Differentiating among these levels is crucial to align the theoretical framing of the study with its empirical focus on student-reported perceptions and institutional accountability. A synthesis of prior empirical studies on Metaverse adoption in education (summarized in Table 1) reveals the dominance of TAM- and UTAUT-based models, alongside notable gaps in sustainability, inclusivity, and governance integration.

**Table 1:** Empirical studies on Metaverse adoption in education.

Study	Context	Theory/Model	Key findings	Limitations
Sunardi et al. (2022)	AR in video conferencing (COVID-19)	UTAUT2	Performance expectancy and facilitating conditions predicted adoption	Limited to pandemic-driven contexts
Yang et al. (2022)	Metaverse in basketball learning	UTAUT2	Social influence and hedonic motivation are critical	Narrow domain, lacks sustainability factors
Maghaydah et al. (2024)	VR-based education satisfaction	TAM + SDT + IS success model	Self-determination is linked to satisfaction	No governance/ESG consideration
Teng et al. (2022)	Educational Metaverse adoption	Extended UTAUT	Trust and perceived interactivity are crucial	Pilot study, small sample
Alawadhi et al. (2022)	Metaverse in medical training	TAM + DOI	Perceived usefulness and innovativeness are significant	Excludes equity and inclusivity
Al-Adwan et al. (2023)	Higher education Metaverse intention	TAM	Perceptions strongly predicted intention	Lacks ESG and policy alignment

**Figure 1:**
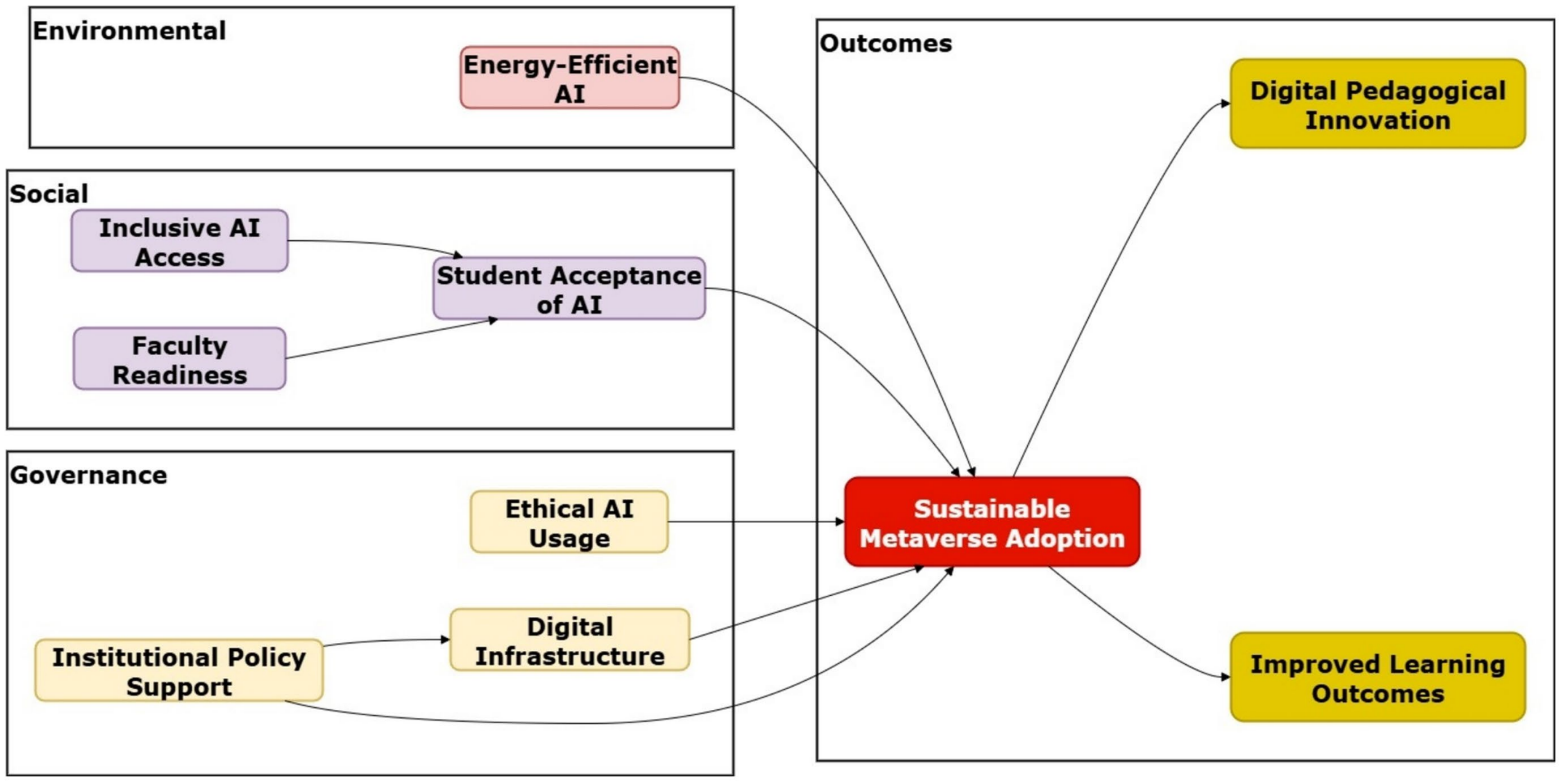
Our proposed research model.

To fill these gaps, this study proposes an ESG-sensitive framework for AI Metaverse adoption in higher education (Figure 1). The model proposed must support environmental sustainability (energy-efficient AI systems and low-carbon digital infrastructure) (Strubell et al., 2020; Shkabatur et al., 2022); social inclusion (equitable access, culture-sensitive, student well-being) (Ul Hassan et al., 2025; Lapidot-Lefler, 2025); and governance and institutional support (ethical AI use, leadership accountability, policy alignment) (Zou et al., 2024; Mohamed Hashim et al., 2022). Moreover, it included technological trust and security, which represent the perceived safety of data and reliability of systems, as these have been shown to influence student participation (Strubell et al., 2020; Jobin et al., 2019). The outcome constructs relate to digital pedagogical innovation (DPI) and learning outcomes, indicating relative advantage and long-term educational value (Chamola et al., 2025; Alfaisal et al., 2024; Grant and Eynon, 2017). Studies reviewed globally highlight three major gaps: the lack of integration of environmental greenness and governance factors into adoption models, a lack of attention to inclusiveness, as immersive technology may increase the digital divide, and heavy reliance on small pilots without robust outcome measurement. To address these gaps, an integrative model that combines established adoption constructs with ESG is needed to support responsible, scalable, and sustainable AI–Metaverse implementation in HEIs.

**Figure 2:**
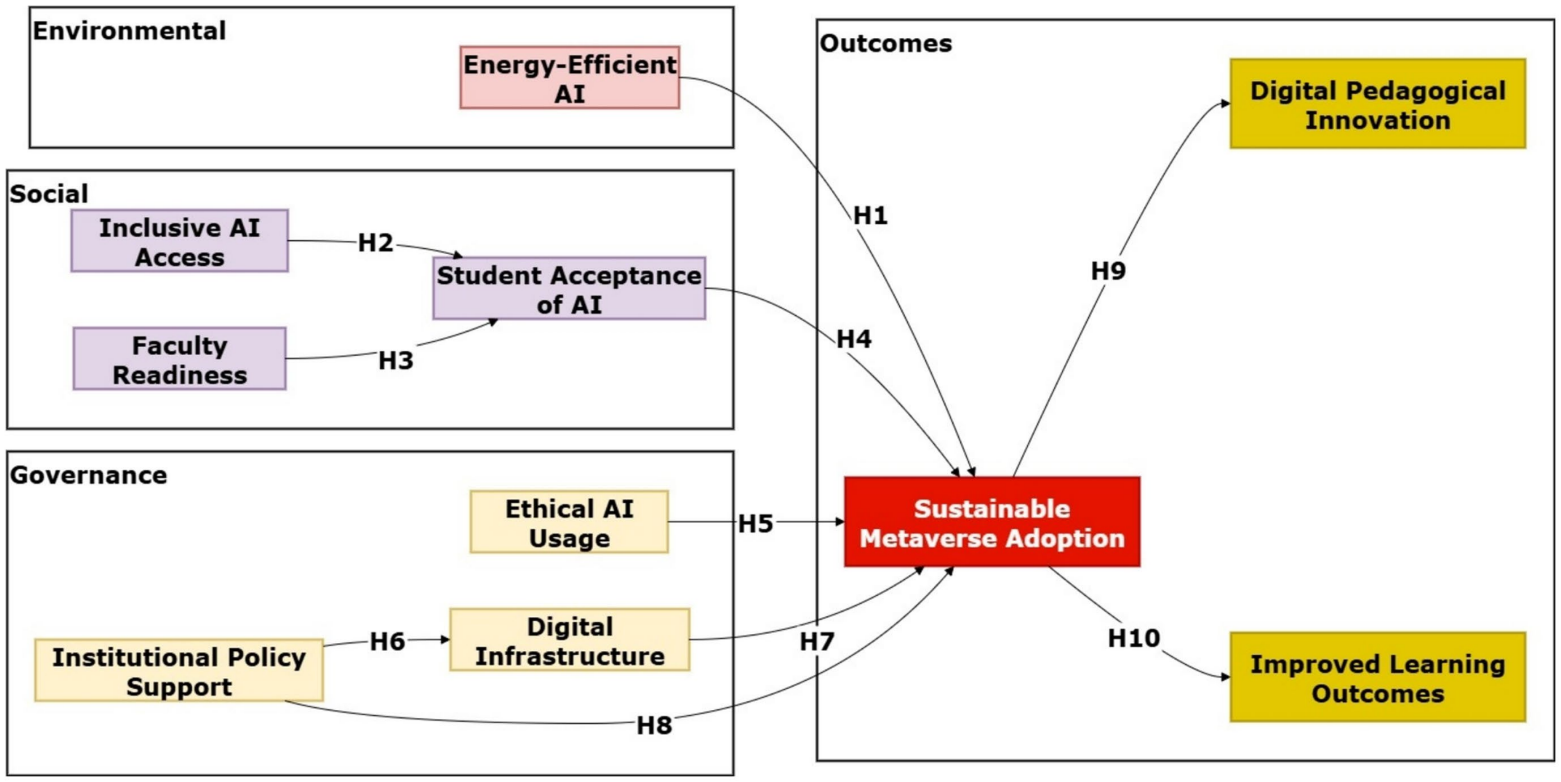
Theoretical research model.

The research hypotheses pertain to the ESG aspects of sustainability, emphasizing their applicability in integrating AI and the Metaverse in higher education. Both hypotheses are grounded in theoretical perspectives, research evidence from studies, and the wider body of literature on sustainable digital transformation, as shown in Figure 2.

The environmental dimension brings the consequences of the large-scale adoption of AI and the Metaverse to focus. Training highly powerful AI models demands substantial computational power, which can lead to high energy consumption and carbon emissions (Strubell et al., 2020). Installing AI-based Metaverse applications, such as immersive simulation applications, intelligent tutoring software, and personal analytical software in educational institutions creates sustainability risks unless educational institutions install energy-efficient infrastructures. Green data centers that are algorithmically designed data centers and renewable-energy-powered cloud infrastructures directly reduce environmental impacts while instilling sustainability measures in education entities (Bawden and Robinson, 2022). Verifying these activities’ congruence with the United Nations’ Sustainable Development Goal (SDG 13: Climate Action) prevents the trade-off between environmental responsibility and educational innovation. Under this context, energy-efficient AI models (EEAI) will help institutions integrate AI-Metaverse technologies sustainably (SAAM) and innovate while fulfilling global climate goals.

*H1*: Energy-Efficient AI Systems (EEAI) positively influence the Sustainable Adoption of the Metaverse (SAAM).

The social aspect of the proposed model highlights inclusivity, fairness, and human preparedness for digital transformation and usage. Inclusive AI Access (IAIA) lies at the heart of making students and educators from all socioeconomic levels benefit equally from AI–Metaverse developments. Beaudry et al. (2019) indicate that exclusionary access exacerbates the digital divide by restraining participation and diminishing legitimacy in digital transformation. Conversely, inclusive design and accessible access enhance fairness, confidence, and acceptance, making institutional take-up more stable (Harris et al., 2023; Zallio and Clarkson, 2021). Another factor, Faculty Readiness (FR), also affects the success of immersive take-up. Teaching staff play a gateway role in introducing AI-supported pedagogic strategies, and their digital fluency, confidence, and pedagogic openness play a decisive role in the take-up of institutional outcomes (Tondeur et al., 2017). Empirical research (Creswell, 2013) verifies that building capacities and recurrent professional training considerably raise the faculties’ use of new technologies. No less important is Student Acceptance of the Metaverse in Academia (SAMA). Students’ perceptions of usefulness, confidence, and engagement strongly shape their willingness to adopt immersive environments. Earlier research indicates that AR/VR and AI tools improve collaborative learning, enthusiasm, and motivation (Hair and Alamer, 2022). In this research, SAMA serves as both a driver and a mediator, explaining how individual perceptions and faculty preparedness correspond to institutional take-up. All these factors emphasize that stable take-up requires a socially inclusive policy that harmonizes students’ expectations and faculties’ competence vis-à-vis equitable access.

*H2*: Inclusive AI Access (IAIA) positively influences the Sustainable Adoption of the Metaverse (SAAM).

*H3*: Faculty Readiness (FR) positively influences the Sustainable Adoption of the Metaverse (SAAM).

*H4*: Student Acceptance of the Metaverse in Academia (SAMA) positively influences the Sustainable Adoption of the Metaverse (SAAM).

Governance factors outline the ethical, strategic, and institutional facets related to technology adoption (Huda, 2019). Kaddoura and Al Husseiny (2023) emphasized the ethical deployment of technologies in the Metaverse, particularly in educational applications that aggregate individual student data and employ algorithmic processing that could introduce biases. Compliance with ethical mandates like transparency, fairness, accountability, and privacy protection supports user trust and institutional credibility (Kaddoura and Al Husseiny, 2023; Hair et al., 2017). Hence, organizations that adhere to ethical governance models derive greater benefits in sustainable and socially responsible technology adoption. Institutional Policy Support (IPS) encompasses two tasks. It influences Digital Infrastructure (DI) by enhancing infrastructure readiness, prescribing governance models, and aligning national strategies with digital transformation processes (Soomro et al., 2024; Preston and Colman, 2000). For example, Saudi Vision 2030 exemplifies that policy dictates teaching technology development (Alhumaid et al., 2023; Etikan et al., 2016). Furthermore, IPS supports sustainable technology integration by specifying governance models, defining accountability measures, and adherence to environmental, societal, and governance (ESG) targets (Alhumaid et al., 2023; Etikan et al., 2016). Digital infrastructure’s role in the adoption process cannot be overstated. It is difficult to integrate technology into teaching efficiently and sustainably in the absence of adequate connectivity, hardware, and secure systems, regardless of existing policies or user readiness (Kourtesis, 2024). Such governance models ensure that new technology is incorporated into teaching settings in ways that make it feasible, ethical, and prudent.

*H5*: Ethical AI Use (EAIU) positively influences the Sustainable Adoption of the Metaverse (SAAM).

*H6*: Institutional Policy Support (IPS) positively influences Digital Infrastructure (DI).

*H7*: Digital Infrastructure (DI) positively influences the Sustainable Adoption of the Metaverse (SAAM).

*H8*: Institutional Policy Support (IPS) positively influences the Sustainable Adoption of the Metaverse (SAAM).

The outcome dimension reveals the impact of education and institutions on the adoption of sustainable technologies. Applications based on the Metaverse, if undertaken in a sustainable and transparent profile, have the potential to bolster DPI. It encompasses the renovation and evolution of teaching methods and learning schemes through virtual simulations, a personalized assessment framework, and virtual collaborative communities (Kline, 2015; Podsakoff et al., 2003). Furthermore, sustainable adoption also correlates with Enhanced Student Learning Outcomes (ESLO). Empirical research has revealed that interactive and immersive learning experiences have a considerable impact on students’ cognitive processes, socialization, and knowledge retention (Hair et al., 2021; Dahri et al., 2024a). These results strongly suggest the adoption of Metaverse-based learning portals in tertiary learning, especially since they optimize learning efficiency while keeping to environmental, societal, and governance (ESG) principles.

*H9*: Sustainable Adoption of the Metaverse (SAAM) positively influences Digital Pedagogical Innovation (DPI).

*H10*: Sustainable Adoption of the Metaverse (SAAM) positively influences Enhanced Student Learning Outcomes (ESLO).

Such a hypothesis development framework frames Metaverse adoption in ESG-based contexts in a manner that environmental concern, social equity, and governance accountability all spearhead digital transformation and superior learning results in university settings.

## 3 Research methodology

This subsection describes the methodological procedures followed in the current study, namely research design, instrument construction, scale of measurement, target population and sample, data collection, data analysis, and ethical issues. A quantitative research approach using a structured survey research design has been followed, and the data have been analyzed using SEM by SmartPLS 4 to ensure the soundness of the research methodology and its consistency with the research goals.

### 3.1 Research design

The research employed a quantitative, cross-sectional design to empirically explore the adoption of Metaverse technologies embedded into AI in higher education using the frameworks of ESG. Quantitative methods are widely assumed to be able to operationalize relationships between constructs in a highly structured and objective manner (Creswell, 2013). With the use of SEM using SmartPLS 4, this research permits the testing of highly complex models in a single step while testing both indirect and direct effects, which is especially appropriate for theory testing and predictive analysis (Hair and Alamer, 2022; Hair Joe et al., 2016). We considered the use of PLS-SEM to be judicious given its strength for predictive modeling. This tool is more appropriate for exploratory research for new technologies like the Metaverse and its acceptability for relatively small to medium sample sizes when contrasted against covariance-based SEM (Hair et al., 2019a; Ringle and Sarstedt, 2016).

### 3.2 Instrument development and validation

The survey questionnaire designed for this study was constructed using validated scales from previous existing studies. We updated and modified the questionnaire in the context of ESG-conscious Metaverse adoption. Following Nunnally and Bernstein (1994), constructs were defined methodically to significantly cover the content area. Items were adopted and modified from authorized sources, such as TAM, and recent Metaverse in education studies (Alfaisal et al., 2024; Maghaydah et al., 2024; Roy et al., 2023; Dhingra and Abhishek, 2024). We engaged experts to review the content validity of the questionnaire. Three education technology experts and ESG frameworks familiarized themselves with the instrument to assess the clarity, relevance, and representativeness of items. Based on their recommendations, ambiguous items were rewritten, and redundant items were removed. To establish reliability and validity, a pilot study with 55 participants (Saudi Arabian university students and teachers well-versed in digital technologies) assessed reliability using Cronbach’s alpha and composite reliability measures, where all the constructs were above the recommended 0.70 threshold level (Hair et al., 2017; Soomro et al., 2024). Construct validity was established by performing exploratory factor analysis, where acceptable loadings and convergent validity (AVE > 0.50) were ensured. Discriminant validity checking using the Fornell–Larcker and HTMT criteria provided further evidence. A 5-point Likert scale for all items (range 1 = strongly disagree to 5 = strongly agree) was utilized. A 5-point scale was chosen to obtain subtle variations in the response of the participants, avoid central tendency bias, and increase reliability. This technique has been highly advocated for technology adoption and SEM investigations for enhanced sensitivity of the scale (Preston and Colman, 2000).

### 3.3 Target population and sampling

The population of interest included higher education students in Saudi Arabia who were experienced or were familiar with the use of AI-based or Metaverse-enabled learning technologies. The Saudi Arabian research context was selected because the nation has a vision for quick digital transformation (Vision 2030) and rapid investment in AI and virtual technologies across the education continuum (Alhumaid et al., 2023). A purposive sampling design was utilized to guarantee that participants have related experience and knowledge regarding emerging education technologies (Etikan et al., 2016). Using the software package G*Power 3.1, the required minimum sample size was estimated using the parameters as follows: effect size f^2^ = 0.15 (medium), *α* = 0.05, power = 0.95, and 10 predictor variables. The simulation indicated a minimum sample of 172 respondents. For purposes of higher robustness and the possibility of excluding values in the event of missing values, 300 questionnaires were administered, and 256 valid cases were accepted for final analysis and met the required threshold for analysis of the SEM (Kline, 2015).

### 3.4 Data collection process

We collected data using a Google Form and distributed the link in online classes and through institutional mailing lists. Learning management software and research networks were used to distribute the survey link after obtaining the consent of the appropriate university departments. The response was voluntary, and informed consent occurred before commencing the survey. The total duration of data collection was 6 weeks, during which reminders were issued periodically to maximize response rates. We applied common procedural remedies for method bias, such as responding anonymously, counterbalancing the ordering of questions, and the use of both positively and negatively phrased items (Podsakoff et al., 2003).

### 3.5 Data analysis process

Data was analyzed using Partial Least Squares SEM, also known by the abbreviation PLS-SEM, through the application SmartPLS4 (Hair and Alamer, 2022). SEM analysis was performed in two steps: (1) In the first step, measurement model evaluation was undertaken, which entailed examining several aspects of reliability, such as Cronbach’s alpha and composite reliability, in addition to examining convergent validity via average variance extracted, also referred to by its abbreviation AVE, and discriminant validity using methods such as Fornell–Larcker and HTMT. (2) In the second step, structural model evaluation was undertaken, in which path coefficients, t-values, and *p*-values were carefully determined via the process of bootstrapping with a total number of 5,000 resamples. In addition to several effect sizes in the form of f^2^, several others were also presented to thoroughly evaluate the model’s ability to explain. This meticulous two-step process ensured that both the quality of the measurement and the rigor of theoretical testing were duly validated. Ethical Considerations: The research adhered to ethical guidelines throughout its design and conduct. Ethical clearance was obtained from the Institutional Review Board of the university that hosts the research scholars. The participants were fully informed about the purpose of the study, the voluntary nature of participation, confidentiality, and their entitlement to withdraw at any point in time without penalty. No personally identifiable information was collected, and the data were stored securely in password-protected files accessible only to the research team. The results are presented in aggregate format so that the identity of the participants remains anonymous and their privacy is preserved.

## 4 Results

### 4.1 Demographic profile of respondents (*n* = 280)

The final sample comprised 280 respondents, with a near-balanced gender distribution. As shown in Table 2, male students accounted for 153 respondents (54.6%), while female students comprised 127 respondents (45.4%), indicating an overall gender-balanced sample appropriate for comparative analysis. In terms of age, the majority of respondents fell within the 21–25 years age group (*n* = 163, 58.2%), which is typical of undergraduate and early postgraduate cohorts. This was followed by participants aged 26–30 years (*n* = 61, 21.8%), those below 20 years (*n* = 36, 12.9%), and those above 30 years (*n* = 20, 7.1%). This age distribution reflects a population predominantly composed of early-career learners, who are commonly identified as early adopters of emerging digital learning technologies. Regarding academic level, the majority of participants were undergraduate students (*n* = 175, 62.5%), followed by master’s students (*n* = 72, 25.7%) and PhD candidates (*n* = 33, 11.8%). This distribution aligns with the age profile and supports the study’s focus on student adoption of AI–Metaverse environments in mainstream higher education contexts. Participants represented a broad range of academic disciplines. Science and engineering students constituted the largest group (*n* = 112, 40.0%), followed by social sciences (*n* = 93, 33.2%), humanities (*n* = 52, 18.6%), and other disciplines (*n* = 23, 8.2%). With respect to prior technological exposure, 201 respondents (71.8%) reported having previously used AI-based learning tools or AI-enabled Metaverse environments, whereas 79 respondents (28.2%) indicated no prior experience. This high level of baseline exposure suggests that the sample was sufficiently familiar with AI–Metaverse technologies, providing a suitable context for examining determinants of sustainable adoption.

**Table 2:** Demographic characteristics of respondents (*N* = 280).

Variable	Category	Frequency (*N*)	Percentage (%)
Gender	Male	153	54.6
	Female	127	45.4
Age	Below 20	36	12.9
	21–25	163	58.2
	26–30	61	21.8
	Above 30	20	7.1
Field of study	Science/Engineering	112	40.0
	Social Sciences	93	33.2
	Humanities	52	18.6
	Other	23	8.2
Level of study	Undergraduate	175	62.5
	Master’s	72	25.7
	PhD	33	11.8
Have used AI tools in learning (AI–Metaverse)	Yes	201	71.8
	No	79	28.2

### 4.2 Preliminary data screening and readiness for SEM

The sample data were filtered for response quality and completeness before the model estimation. The missing data were low (<2%) and random across items, as evidenced by the absence of systematic patterns when inspected across cases and across variables. These items were free of extreme univariate outliers (|z| > 3.29). At the indicator level (outer model) and the construct level (inner model), multicollinearity diagnostics were performed according to the recommendations of PLS-SEM. The variance inflation factors (VIFs) were under the conservative threshold of 3 at the indicator level. This indicates no problematic collinearity among the observed measures. Likewise, the inner VIF values for structural model constructs were also below recommended cut-offs, revealing no multicollinearity among predictor constructs (Hair et al., 2021). When it comes to sample adequacy, the final sample size of our study (*N* = 280) exceeds the minimum PLS-SEM requirements on account of the model’s complexity, number of predictors, and statistical power considerations, and is a lot more than the 10-times rule as well as recent power-based recommendations for structural models of similar complexity. The data quality was adequate, the measurement properties showed strong reliability and convergent validity above recommended thresholds, and the sample sizes were adequate. Overall, the suitability of the dataset for measurement and structural model estimation using SmartPLS 4 (Hair et al., 2017).

### 4.3 Measurement model – factor loadings and multicollinearity assessment

As presented in Table 3, the indicator loadings and variance inflation factor (VIF) values are all constructs in the measurement model. Reflective measures were used to estimate the constructs because all of them are reflective, consistent with earlier technology adoption and sustainability literature. The reflective measures were evaluated using indicator loadings, the composite reliability, average variance extracted (AVE), and collinearity diagnostics. According to Hair et al. (2017), item loadings above 0.70 are accepted and indicate good reliability of items. All indicators, as presented in Table 3, have loadings of 0.5 or greater. This confirms that the items are reliable and that the constructs are adequately convergent. The loadings indicate that the variance shared between each indicator and its corresponding latent construct is substantial and consistent with the theoretical specification in the measurement model. The variables Digital Infrastructure (DI), Faculty Readiness (FR), Student Acceptance of AI (SAAM), Sustainable AI−Metaverse Adoption (SAMA), and Inclusive AI Access (IAIA) have high loadings (≥ 0.75), indicating the convergence of their underlying variables. Similarly, EAIU, ESLO, DPI, and EEAI exhibit good and stable loadings, as corroborated in past studies, including research on digital learning, sustainable development, educational technology, innovation, and robotics (Hair et al., 2019a; Dahri et al., 2024a). Further, the results of the collinearity diagnostics support the adequacy of the measurement model. The range of all the VIF values is from 1.36 to 2.82, which is below the conservative threshold of 3.3. Thus, multicollinearity is not an issue between the indicators (Hair and Alamer, 2022; Diamantopoulos and Siguaw, 2006). This confirms that the indicators are non-redundant and that each construct taps a different conceptual domain. Overall, the higher factor loadings indicated that these reflective constructs had confirmed convergent validity. Also, the VIF values showed no multicollinearity in the measurement model. These results justify the continued use of each indicator in assessing composite reliability, average variance extracted (AVE), discriminant validity (HTMT), and structural model using SmartPLS 4.

**Table 3:** Factor loadings and VIF values.

Constructs	Code	Items	Loadings	VIF
Digital Infrastructure (DI)	DI01	I have good internet access to use AI-powered tools.	0.768	1.619
	DI02	My university provides access to modern digital tools and systems.	0.83	1.847
	DI03	I feel the digital environment in my institution supports the use of AI technologies.	0.835	1.933
	DI04	I find it easy to use AI tools due to the available infrastructure.	0.774	1.555
Digital Pedagogical Innovation (DPI)	DPI01	I think AI and the Metaverse support innovative teaching methods.	0.816	1.795
	DPI02	I believe these tools can help instructors create engaging learning environments.	0.803	1.722
	DPI03	I feel AI/Metaverse enables new ways of interaction and collaboration.	0.841	2.017
	DPI04	I believe digital tools improve creativity in teaching and learning.	0.803	1.68
Ethical AI Usage (EAIU)	EAIU01	I think AI tools should adhere to ethical guidelines in education.	0.768	1.518
	EAIU02	I worry about how AI handles student data and privacy.	0.779	1.674
	EAIU03	I prefer AI systems that explain their operation.	0.846	1.865
	EAIU04	I trust AI systems more when they are transparent and accountable.	0.810	1.758
Energy-Efficient AI Systems (EEAI)	EEAI01	EEAI1: I believe AI tools in education should use minimal energy resources.	0.853	1.37
	EEAI02	EEAI2: I am more likely to support AI if it is environmentally friendly.	0.859	2.182
	EEAI03	I care about the carbon footprint of AI technologies used in education.	0.854	2.079
	EEAI04	I prefer digital tools that contribute to environmental sustainability.	0.797	1.666
Enhanced Student Learning Outcomes (ESLO)	ESLO01	I believe AI tools improve my understanding of the subject.	0.771	1.52
	ESLO02	I feel AI helps me perform better in assessments and assignments.	0.823	1.785
	ESLO03	I believe Metaverse tools increase my motivation to learn.	0.788	1.637
	ESLO04	I feel I learn more effectively using AI and Metaverse tools.	0.81	1.746
Faculty Readiness (FR)	FR01	My instructors are confident in their use of AI-based tools.	0.815	1.961
	FR02	I believe faculty members receive training on AI tools.	0.799	1.892
	FR03	Faculty members effectively integrate AI into classroom teaching.	0.805	1.938
	FR04	I can ask teachers for help in using AI-based learning tools.	0.802	1.869
	FR05	My teachers actively promote the use of AI in learning activities.	0.734	1.617
Inclusive AI Access (IAIA)	IAIA01	I believe all students should have equal access to AI-based learning tools.	0.79	1.779
	IAIA02	I feel AI tools should support students with diverse learning needs.	0.848	2.125
	IAIA03	I support the idea that AI should be designed to accommodate students with different skill levels.	0.854	2.207
	IAIA04	I think AI in education should be accessible regardless of socio-economic background.	0.848	1.994
Institutional Policy Support (IPS)	IPS01	I believe my university has clear policies governing the use of AI in teaching and learning.	0.804	1.489
	IPS02	I am aware of the guidelines regarding the ethical use of AI in my institution.	0.807	1.536
	IPS03	I feel that my institution encourages the use of AI in a responsible way.	0.856	1.623
Student Acceptance of AI (SAAM)	SAAM01	I enjoy using AI tools in my learning.	0.829	2.346
	SAAM02	I find AI-based tools useful for improving my academic performance.	0.869	2.822
	SAAM03	I am willing to continue using AI tools for my studies.	0.816	2.086
	SAAM04	I believe AI tools improve my learning experience.	0.823	2.067
	SAAM05	I feel comfortable using AI technologies in education.	0.819	2.044
Sustainable AI-Metaverse Adoption (SAMA)	SAMA01	I support the long-term use of AI and the Metaverse in education.	0.789	1.806
	SAMA02	I believe using AI-Metaverse can make education more future-ready.	0.829	2.091
	SAMA03	I am interested in using AI and Metaverse tools regularly in learning.	0.856	2.535
	SAMA04	I believe AI-Metaverse tools should be integrated into higher education.	0.821	2.12
	SAMA05	I see the use of AI-Metaverse tools as a sustainable educational solution.	0.702	1.493

To assess internal consistency and convergent validity, Cronbach’s alpha, composite reliability (CR), and average variance extracted (AVE) were evaluated, as summarized in Table 4. Although composite reliability is the preferred reliability measure in PLS-SEM due to its ability to account for differing indicator loadings, Cronbach’s alpha was also reported as a conservative lower-bound estimate of internal consistency, consistent with established practice in PLS-SEM studies (Hair et al., 2019b; Soomro et al., 2025). Cronbach’s alpha values for all constructs ranged from 0.762 to 0.888, exceeding the recommended threshold of 0.70 (Nunnally and Bernstein, 1994), thereby indicating satisfactory internal consistency. Composite reliability values ranged from 0.863 to 0.918, all well above the recommended minimum of 0.70 (Hair et al., 2019b; Al-Rahmi et al., 2026), providing strong evidence of construct reliability and confirming the stability of the reflective measurement model. With respect to convergent validity, AVE values ranged between 0.626 and 0.698, exceeding the minimum acceptability threshold of 0.50 (Fornell and Larcker, 1981). These results indicate that each construct explains more than 50% of the variance in its indicators, thereby establishing adequate convergent validity. Cronbach’s alpha provides a conservative assessment of internal consistency; the high composite reliability and AVE values serve as the primary evidence supporting the reliability and convergent validity of the constructs. Collectively, these findings confirm the robustness and soundness of the reflective measurement model and justify its suitability for subsequent structural model analysis in SmartPLS 4.

**Table 4:** Internal consistency and convergent validity.

Variables	Cronbach’s alpha	Composite reliability	Average variance extracted (AVE)
DI	0.8150	0.8780	0.6440
DPI	0.8320	0.8880	0.6660
EAIU	0.8150	0.8780	0.6420
EEAI	0.8160	0.8790	0.6470
ESLO	0.8100	0.8750	0.6370
FR	0.8510	0.8930	0.6260
IAIA	0.8560	0.9020	0.6980
IPS	0.7620	0.8630	0.6770
SAAM	0.8880	0.9180	0.6910
SAMA	0.8590	0.8990	0.6420

Discriminant validity was evaluated according to the heterotrait–monotrait (HTMT) ratio and the Fornell–Larcker criterion for reflective measurement models in PLS-SEM (see Tables 5, 6). The HTMT values of all constructs were below the conservative threshold of 0.85 and the liberal threshold of 0.90, which implies their empirical distinctiveness (Henseler et al., 2015). The HTMT values ranged from 0.474 to 0.896. The HTMT values for Digital Infrastructure (DI) and Faculty Readiness (FR) are high but less than the upper bound value of 0.90. Thus, this is not a violation of discriminant validity. It is theoretically feasible for the institutional digital infrastructure to enable and support faculty readiness, particularly in technology-enabled learning environments. But value does not equal measurement redundancy. As shown in Table 5, all HTMT values were assessed against the threshold values and were found to be within limits. Based on HTMT, discriminant validity is adequate as no construct pair exceeded the recommended cut-off values.

**Table 5:** HTMT analysis.

	DI	DPI	EAIU	EEAI	ESLO	FR	IAIA	IPS	SAAM	SAMA
DI										
DPI	0.661									
EAIU	0.631	0.632								
EEAI	0.544	0.514	0.474							
ESLO	0.81	0.652	0.536	0.658						
FR	0.896	0.67	0.563	0.672	0.861					
IAIA	0.574	0.505	0.572	0.806	0.693	0.696				
IPS	0.634	0.642	0.833	0.491	0.558	0.58	0.563			
SAAM	0.689	0.68	0.625	0.612	0.818	0.839	0.719	0.629		
SAMA	0.519	0.52	0.761	0.685	0.718	0.736	0.718	0.761	0.719	

**Table 6:** Fornell–Larcker criterion.

	DI	DPI	EAIU	EEAI	ESLO	FR	IAIA	IPS	SAAM	SAMA	
DI	0.802										
DPI	0.547	0.816									
EAIU	0.516	0.521	0.801								
EEAI	0.447	0.426	0.385	0.804							
ESLO	0.739	0.536	0.439	0.535	0.798						
FR	0.757	0.565	0.475	0.561	0.964	0.791					
IAIA	0.481	0.426	0.476	0.669	0.579	0.597	0.835				
IPS	0.502	0.513	0.971	0.386	0.44	0.47	0.452	0.823			
SAAM	0.589	0.586	0.536	0.524	0.695	0.733	0.628	0.521	0.831		
SAMA	0.437	0.441	0.647	0.565	0.598	0.631	0.616	0.625	0.632	0.801	

The Fornell–Larcker criterion was used to evaluate discriminant validity. The square root AVE values (diagonal elements) were greater than the inter-construct correlations for all constructs in the rows and columns. This confirms that each construct shares more variance with its indicators than with other constructs. The results from both HTMT and the Fornell–Larcker criterion, combined, support the measurement model’s conformity to the standard of discriminant validity. Each construct encapsulates a unique conceptual realm in the proposed ESG-informed AI–Metaverse adoption framework, indicating the robustness of the measurement model for the structural model analysis.

### 4.4 Structural model assessment

According to PLS-SEM guidelines Hair et al. (2019a), the performance assessment of the structural model employed the coefficient of determination (R^2^) and effect sizes (f^2^). The outcomes suggest that the key endogenous constructs such as Digital Infrastructure (DI), Student Acceptance of AI (SAAM), Sustainable AI–Metaverse Adoption (SAMA), and ESLO possess satisfactory explanatory power. The model can explain 25.2% of the DI variance (R^2^ = 0.252), indicating institutional policy support meaningfully explains infrastructure readiness. SAMA’s R^2^ of 0.487 indicates it explains about 49% of the variance in higher education institutions’ readiness for AI integration. The analysis reveals that SAAM has the highest explanatory power (R^2^ = 0.573), with environmental, social, governance, and sustainability-related antecedents jointly accounting for student acceptance of AI. The R^2^ value calculated by ESLO is 0.484. Together, these results demonstrate that the adoption of AI–Metaverse in education has explained almost half of the difficulty of learning outcomes. In general, the R^2^ values are moderately high to high for the key adoption and outcome constructs. The analysis of effect size determines the relative contribution of each exogenous construct to its endogenous variable. Institutional policy support (IPS → DI) produced a medium-to-large effect (f^2^ = 0.337), validating the important role of governance in enabling digital infrastructure. The Faculty Readiness (FR → SAMA) and Inclusive AI Access (IAIA → SAMA) possessed medium effects (f^2^ = 0.210 and 0.175), respectively, which had a significant effect on sustainable adoption. Regarding acceptance by students, Digital Infrastructure (DI → SAAM) has a medium effect size (f^2^ = 0.152). While Sustainable AI – Metaverse Adoption (SAMA→SAAM) has a small-to-medium effect (f^2^ = 0.124), the outcome that measured environmental sustainability through Energy-Efficient AI Systems (EEAI → SAAM) had a small but meaningful effect (f^2^ = 0.029). Conversely, EAIU (Ethical AI Usage) (SAAM) and IPS (Institutional Policy Support) (SAAM) had no direct effects (f ^2^ ≈ 0.00). With respect to the outcomes, SAAM had a strong effect on DPI (SAAM → DPI, f^2^ = 0.522) and a large effect on ESLO (SAAM → ESLO). Despite the high magnitude of the numerical f^2^ value for SAAM → ESLO, it is interpreted conservatively as a large effect, which means that sustained acceptance of AI–Metaverse technologies is a key driver of student learning outcomes.

**Figure 3:**
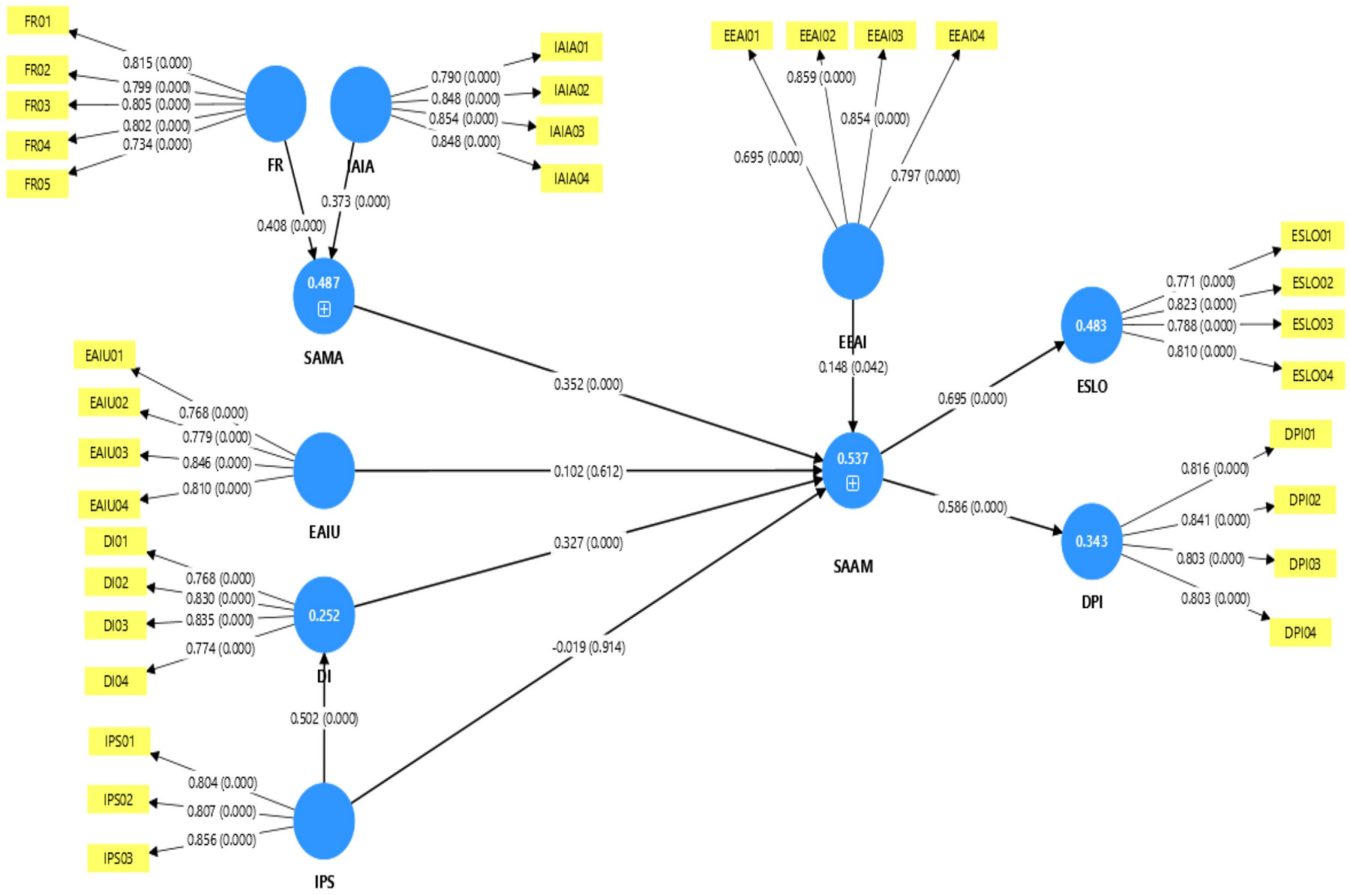
Hypothesis results.

Table 7 reports the direct path estimates for H1–H10. The environmental factor, Energy-Efficient AI Systems (EEAI), positively influenced Sustainable AI–Metaverse Adoption (SAAM) (H1: *β* = 0.148, *p* = 0.042). Within the social dimension, Inclusive AI Access (IAIA) (H2: *β* = 0.373, *p* < 0.001) and Faculty Readiness (FR) (H3: *β* = 0.408, *p* < 0.001) significantly predicted Student Acceptance of AI–Metaverse (SAMA) rather than SAAM directly. In turn, SAMA significantly influenced SAAM (H4: *β* = 0.352, *p* < 0.001), confirming a mediating mechanism. Formal bootstrapped mediation tests showed significant indirect effects for IAIA → SAMA → SAAM and FR → SAMA → SAAM, with non-significant direct paths, indicating full mediation. Governance effects were largely indirect. Ethical AI Usage (EAIU) did not significantly affect SAAM (H5: *β* = 0.102, *p* = 0.612). Institutional Policy Support (IPS) strongly predicted Digital Infrastructure (DI) (H6: *β* = 0.502, *p* < 0.001), and DI significantly enhanced SAAM (H7: *β* = 0.327, *p* < 0.001), while the direct IPS → SAAM path was not supported (H8: *β* = −0.019, *p* = 0.914). Finally, SAAM strongly influenced DPI (H9: *β* = 0.586, *p* < 0.001) and ESLO (H10: *β* = 0.695, *p* < 0.001), confirming robust downstream effects (Figure 3).

**Table 7:** Direct paths.

Hypothesis No.	Structural path	*β*	*t*-value	*p*-value	Result
H1	EEAI → SAAM	0.148	2.038	0.042	Supported
H2	IAIA → SAMA	0.373	5.453	<0.001	Supported
H3	FR → SAMA	0.408	5.670	<0.001	Supported
H4	SAMA → SAAM	0.352	3.546	<0.001	Supported
H5	EAIU → SAAM	0.102	0.507	0.612	Not Supported
H6	IPS → DI	0.502	10.801	<0.001	Supported
H7	DI → SAAM	0.327	5.047	<0.001	Supported
H8	IPS → SAAM	−0.019	0.108	0.914	Not Supported
H9	SAAM → DPI	0.586	11.823	<0.001	Supported
H10	SAAM → ESLO	0.695	15.650	<0.001	Supported

## 5 Discussion

This study examined how ESG dimensions jointly shape Sustainable AI–Metaverse Adoption (SAAM) in higher education and how this adoption translates into DPI and ESLO. Based on sustainability-oriented technology adoption and institutional capability theories (Venkatesh et al., 2012; Dwivedi et al., 2023), the results strongly align with the study objectives and advance theoretical understanding of sustainability-driven AI–Metaverse integration in education.

### 5.1 Environmental dimension: strategic rather than behavioral influence (H1)

The environmental dimension reveals that Energy-Efficient AI Systems (EEAI) positively influence SAAM (H1). Although the effect size is modest, the relationship is statistically significant, indicating that environmental sustainability functions as a strategic legitimacy signal rather than a primary behavioral driver. This finding aligns with green IT and sustainable technology literature, which suggests that energy efficiency contributes more to long-term adoption credibility than to immediate usage decisions (Zou et al., 2024; Mohamed Hashim et al., 2022). From a theoretical perspective, EEAI represents a macro-level ESG attribute, embedded within institutional sustainability narratives rather than day-to-day user interaction. Students may not directly experience energy efficiency during system use, yet its presence reinforces the perception that AI–Metaverse initiatives align with broader environmental responsibilities. Similar patterns have been observed in prior studies where environmental attributes supported adoption continuity rather than initial acceptance (Whittlestone et al., 2019; Tondeur et al., 2017). Thus, EEAI strengthens sustainable adoption indirectly by anchoring AI–Metaverse systems within environmentally responsible institutional strategies.

### 5.2 Social dimension: student acceptance as a mediating mechanism (H2–H4)

The social dimension produces the most theoretically significant insights. Inclusive AI Access (IAIA) and Faculty Readiness (FR) significantly predict Student Acceptance of AI–Metaverse (SAMA) (H2 and H3), yet neither construct directly influences SAAM. Instead, SAMA fully mediates these relationships, with SAMA → SAAM (H4) strongly supported. This pattern underscores the centrality of student acceptance as a psychological and behavioral gateway through which social conditions translate into sustainable adoption. Inclusive access ensures fairness, equity, and usability, reducing structural barriers and reinforcing social legitimacy (Creswell, 2013). Faculty readiness, meanwhile, shapes the instructional climate, quality of guidance, and confidence with which AI–Metaverse tools are introduced. However, consistent with user-centric adoption theories (Davis et al., 1989; Venkatesh, 2000), these conditions alone do not guarantee sustainable adoption unless students themselves internalize them as beneficial and trustworthy. Theoretically, this supports the view that students function as final arbiters of sustainability in educational technology ecosystems. Faculty preparedness and inclusivity act as enabling conditions, but adoption becomes sustainable only when students cognitively and affectively accept AI–Metaverse systems (Al-Rahmi et al., 2022).

### 5.3 Governance dimension: indirect and contextualized effects (H5–H8)

Governance-related findings require careful interpretation. Ethical AI Usage (EAIU) does not significantly influence SAAM (H5), and Institutional Policy Support (IPS) shows no direct effect on SAAM (H8). Rather than challenging ESG theory, these results indicate that governance mechanisms function through layered, indirect, and structural pathways. Ethical AI principles, such as transparency, accountability, and fairness, are typically embedded at institutional or system-design levels. For students, these safeguards are often assumed rather than consciously evaluated, rendering them less salient during everyday use. Prior literature similarly suggests that ethical considerations tend to influence adoption through trust climates, professional norms, and governance legitimacy, rather than direct behavioral intention (Jobin et al., 2019; Musawa et al., 2024). This explains why EAIU remains theoretically important but empirically non-significant at the user level. Conversely, IPS strongly predicts Digital Infrastructure (DI) (H6), which in turn significantly influences SAAM (H7). This confirms that governance operates as an enabling force, shaping adoption through infrastructural investment, platform readiness, and institutional capacity rather than through direct student perception. Consistent with earlier studies (Al-Mamary et al., 2025), institutional policies often remain invisible to students, whose adoption decisions are driven more by system availability and usability than by formal governance frameworks.

### 5.4 Digital infrastructure as the operational backbone of sustainability

The strong and direct effect of DI on SAAM reinforces its role as the material backbone of AI–Metaverse ecosystems. Infrastructure determines reliability, accessibility, bandwidth, and system integration—features that are immediately experienced by users. Without robust infrastructure, even ethically sound and pedagogically innovative systems struggle to achieve sustained use (Ullah et al., 2021). Theoretically, DI bridges governance and user behavior by translating institutional policies into tangible system capabilities. This explains its mediating role between IPS and SAAM and highlights infrastructure as a necessary condition for sustainability-oriented adoption.

### 5.5 Sustainable adoption as a catalyst for pedagogical innovation (H9) and learning outcomes (H10)

One of the most robust findings is the strong influence of SAAM on DPI (H9). This indicates that pedagogical innovation emerges not from short-term experimentation but from stable and sustained adoption. When AI–Metaverse systems become embedded in routine teaching practices, educators are more willing to redesign instructional strategies, assessment models, and interactive learning environment approaches (Illi and Elhassouny, 2025; Mukred et al., 2025; Wang and Huang, 2025). Importantly, DPI reflects practice-based transformation rather than immediate performance outcomes. Innovation evolves through iterative use, experimentation, and normalization, reinforcing the argument that sustainability precedes meaningful pedagogical change. The strongest relationship in the model is between SAAM and ESLO (H10). This confirms that measurable improvements in performance, engagement, and motivation occur only when adoption is sustained over time. Unlike perception-based constructs such as SAMA and SAAM, ESLO represents objective educational outcomes, capturing tangible academic benefits (Dahri et al., 2024c; Dahri et al., 2025a). This distinction is theoretically critical. It prevents over-attribution of learning gains to short-term AI exposure and instead positions learning outcomes as downstream effects of sustained adoption processes mediated by acceptance, infrastructure, and pedagogical innovation.

The findings reveal a hierarchical ESG-driven adoption logic. Environmental and governance factors operate as contextual enablers, social factors act through student acceptance, and digital infrastructure provides the operational foundation. Sustainable adoption then becomes the central mechanism linking ESG conditions to pedagogical innovation and learning outcomes. This integrated perspective advances existing adoption models by demonstrating that sustainability-oriented AI–Metaverse adoption in higher education emerges from interacting structural, social, and technological mechanisms, rather than from any single dominant factor. This study makes three key theoretical contributions. First, it empirically establishes student acceptance (SAMA) as a mediating mechanism linking social conditions to sustainable adoption. Second, it clarifies that ethical AI use and governance exert influence indirectly, challenging assumptions of direct behavioral effects. Third, it distinguishes clearly between perception-based adoption mechanisms and outcome-level educational impacts, offering a process-oriented explanation of how AI–Metaverse adoption translates into pedagogical and learning gains. Overall, the findings confirm that sustainable AI–Metaverse adoption is not merely a technological or ethical issue but a systemic, multi-layered educational transformation process grounded in ESG principles.

### 5.6 Theoretical and practical implications

This research provides a number of theoretical contributions to the educational technology adoption and sustainability literature. First, by incorporating ESG factors into the technology adoption model, it broadens the applicability of models such as the Technology Acceptance Model and the Unified Theory of Acceptance and Use of Technology. Historically, these models have focused on individual perceptions that are positive and easy to use. Yet, our research finds that wider system perspectives, such as energy efficiency and inclusivity, are also crucial for describing patterns of adoption. This adds to the theoretical literature by connecting sustainability frameworks to technology adoption. Furthermore, the factors that are identified as non-significant, for instance, ethical AI use and faculty preparedness, question established wisdom in current literature and indicate that such factors may have a role through indirect mechanisms rather than a mechanism for direct influence. This opens the way to superior models that include mediating or moderating channels and hence invites further research to consider re-specifying the assumed linear adoption models. Moreover, by establishing that sustainable adoption has a direct influence upon DPI and students’ learning outcomes, this research expands the theoretical understanding of adoption beyond just the stage of decision-making, providing empirical evidence that sustained use has long-term educational dividends.

These findings have real-world applications for the adoption of AI–Metaverse technology in higher education by teachers, organizations, and policymakers. With increased student engagement, teachers have played a pivotal role in elevating student involvement considerably. Sustainable adoption depends on student receptivity and teacher innovation readiness. Teachers should develop learning spaces that instill confidence and curiosity in students to adopt AI-enabled media. Digital infrastructure and inclusivity capabilities are center stage to successful adoption in this report. Infrastructure investments in energy-optimized building stock, equal access to AI, and comprehensive training increase institutional readiness. Policymakers require successful policies with adequate infrastructure and student-centric efforts. Top-down technology imposition has the risk of being counterproductive in the absence of capacity-building or inclusivity. The correlation among sustainable adoption, pedagogical innovation, and superior outcomes suggests that AI–Metaverse has the potential to transform teaching practice. It enables interactive learning that stimulates innovation and tangible advantages. Finally, this research guidebook informs higher education on how to align the adoption of AI–Metaverse with education sustainability and effectiveness.

## 6 Conclusion

This study aimed to examine how sustainable adoption of the Metaverse and AI could transform higher education. It established a framework to conduct Sustainable AI–Metaverse Adoption (SAAM) determinants and their impact on DPI and student learning outcomes (ESLO). A cross-sectional questionnaire for university students, combined with PLS-SEM analysis, added empirical and theoretical knowledge of technology adoption in teaching. Energy-efficient AI systems, digital infrastructure, inclusive access, and student acceptance are important drivers of sustainable adoption, while institutional policy supports infrastructure indirectly. The relative insignificance of importance in ethical AI applications suggests that students value ease of access and use at this juncture. SAAM significantly increases DPI and academic performance, confirming that sustainable adoption supports short-term digital readiness and long-term outcomes. This research integrates ESG perceptions into the study of technology adoption, employing a multidimensional comprehension of sustainability in the digital realm. It provides actionable recommendations to educators and policymakers on the importance of infrastructure, equitable access, and student acceptance for successful adoption. Overall, this research concludes that teaching sustainable AI-Metaverse in higher education institutions is inevitable. In line with sustainability, the incorporation of this technology supports innovation, inclusivity, and high-quality learning attainment. Future research must apply this framework to heterogeneous settings and introduce parameters such as generative AI and governance processes.

### 6.1 Limitations and future research directions

This study acknowledges several limitations that open up interesting directions for future research. The use of a cross-sectional survey design in the study limits the ability to make causal inferences on the constructs. The temporal ordering of AI–Metaverse adoption, pedagogical innovation, and learning outcomes, though based on strong theoretical foundations, cannot be conclusively established due to the presence of statistically significant structural paths. For future research, it would be beneficial to adopt longitudinal or panel designs to examine students’ acceptance, institutional readiness, and learning impact over time, given that AI and the Metaverse technologies are evolving rapidly. Furthermore, only higher education students are sampled, which may limit the generalizability of the findings to other levels, cultures, or professional training. Future research should apply the model to other educational settings, including secondary education, vocational education and training, and lifelong learning contexts, and test it across cultures to see if the sustainable AI–Metaverse adoption mechanisms generalize across settings and societies. Finally, while the framework incorporates new environmental social governance measures and technology adoption theories, it does not cover all sustainable adoption drivers at this stage. Future studies could enhance the theoretical model by integrating individual-level psychological constructs, such as digital resilience, self-regulation, intrinsic motivation, or AI self-efficacy, which may better account for variations in adoption and usage behaviors. The fourth point suggests that the non-significant direct effects of ethical AI usage and faculty readiness on sustainable adoption indicate that these governance- and capability-related factors might operate through indirect or conditional mechanisms. Future research should directly investigate mediation, moderation, and multi-group effects. For instance, it may examine whether ethical AI influences use indirectly through trust, perceived legitimacy, and institutional climate, and whether faculty readiness becomes salient under particular infrastructure or policy conditions. From a methodological standpoint, PLS-SEM is appropriate for exploratory and predictive modeling, yet it may oversimplify the higher-order or multilevel institutional dynamics. In the future, triangulation of the current study’s findings using techniques such as covariance-based SEM multilevel modeling or mixed methods is encouraged. Future studies could incorporate qualitative comments from faculty members, administrators, and students. This would offer a deeper understanding of the mechanisms that embed context and governance. The adoption of AI–Metaverse is a fast-evolving technology. In view of the emergence of generative AI, immersive analytics, and ethical governance frameworks, future studies should periodically revise the theory by incorporating these technological affordances, sustainability measures, and regulatory developments to maintain theoretical relevance and applicability.

## Data Availability

The original contributions presented in the study are included in the article/supplementary material, further inquiries can be directed to the corresponding author.
